# MicroRNA-503-3p affects osteogenic differentiation of human adipose-derived stem cells by regulation of Wnt2 and Wnt7b under cyclic strain

**DOI:** 10.1186/s13287-020-01842-0

**Published:** 2020-07-25

**Authors:** Yadong Luo, Xu Ding, Huan Ji, Meng Li, Haiyang Song, Sheng Li, Chenxing Wang, Heming Wu, Hongming Du

**Affiliations:** 1 Department of Oral and Maxillofacial Surgery, Affiliated Stomatological Hospital of Nanjing Medical University, Hanzhong Road No.136, Nanjing, 210029 Jiangsu Province People’s Republic of China; 2 Jiangsu Key Laboratory of Oral Disease, Nanjing Medical University, Nanjing, 210029 Jiangsu Province People’s Republic of China

**Keywords:** hASCs, Cyclic strain, Osteogenic differentiation, miR-503-3p, Wnt2, Wnt7b

## Abstract

**Background:**

MicroRNAs (miRNAs) play a role in regulating osteogenic differentiation (OD) of mesenchymal stem cells by inhibiting mRNAs translation under cyclic strain. miR-503-3p was downregulated in OD of human adipose-derived stem cells (hASCs) in vivo under cyclic strain in our previous study, while it might target the Wnt/β-catenin (W-β) pathway. In this study, we explored miR-503-3p’s role in OD of hASCs under cyclic strain.

**Methods:**

OD of hASCs was induced by cyclic strain. Bioinformatic and dual luciferase analyses were used to confirm the relationship between Wnt2/Wnt7b and miR-503-3p. Immunofluorescence was used to detect the effect of miR-503-3p on Wnt2/Wnt7b and β-catenin in hASCs transfected with miR-503-3p mimic and inhibitor. Mimic, inhibitor, and small interfering RNA (siRNA) transfected in hASCs to against Wnt2 and Wnt7b. Quantitative real-time PCR (RT-PCR) and western blot were used to examine the OD and W-β pathway at the mRNA and protein levels, respectively. Immunofluorescence was performed to locate β-catenin. ALP activity and calcium were detected by colorimetric assay.

**Results:**

Results of immunophenotypes by flow cytometry and multi-lineage potential confirmed that the cultured cells were hASCs. Results of luciferase reporter assay indicated that miR-503-3p could regulate the expression levels of Wnt2 and Wnt7b by targeting their respective 3′-untranslated region (UTR). Under cyclic strain, gain- or loss-function of miR-503-3p studies by mimic and inhibitor revealed that decreasing expression of miR-503-3p could significantly bring about promotion of OD of hASCs, whereas increased expression of miR-503-3p inhibited OD. Furthermore, miR-503-3p high-expression reduced the activity of the W-β pathway, as indicated by lowering expression of Wnt2 and Wnt7b, inactive β-catenin in miR-503-3p-treated hASCs. By contrast, miR-503-3p inhibition activated the W-β pathway.

**Conclusions:**

Collectively, our findings indicate that miR-503-3p is a negative factor in regulating W-β pathway by Wnt2 and Wnt7b, which inhibit the OD of hASCs under cyclic strain.

## Introduction

In 2001, hASCs were extracted for the first time by digestion of human adipose tissue [1]. hASCs have extensive proliferative potential and the ability to differentiate toward adipogenic, osteogenic, chondrogenic, and myogenic lineages [2, 3]. Compared with bone mesenchymal stem cells, that are normally utilized as seed cells in bone tissue engineering, the advantages of hASCs include a large number of cell sources, easy accession, and rapid proliferation [4]. More importantly, the osteogenic differentiation (OD) activity of ASCs does not decrease with the increase of donor age [5]. Current studies suggest that hASCs may be an important new source of seed cells in bone tissue engineering. An important aspect of bone regeneration that requires further study is to determine how in vitro OD of hASCs can be effectively promoted [6–8]. Furthermore, studies indicate that tensile strain can effectively promote OD of hASCs in vitro [6, 9]; these results have been used to promote OD in bone regeneration [6–8].

The Wnt signaling pathway plays significant roles in regulating many vital biological processes, like embryonic formation and development, stem cells differentiation and maintenance [10, 11]. The W-β signaling pathway plays an essential role in OD of stem cells. The activated W-β pathway can bring about upregulation of specific genes for OD, such as runt-related transcription factor 2 (RUNX2), distal-less homeobox 5, thereby promoting OD [12]. At the same time, activation of the W-β pathway also plays a role in inhibition of adipogenic differentiation of stem cells [13, 14]. Our previous experiments confirm that the W-β signaling pathway could be activated in the process of OD of hASCs induced by cyclic tensile strain [6].

miRNAs, which are non-coding small RNAs, have the ability to bring about inhibition of the expression of target genes by suppressing the translation or degradation of target mRNAs. They play an essential role in human physiological and pathological processes, including apoptosis, cell division, differentiation, and organ development [15–17]. The previous report had indicated that the process of OD regulated by miRNAs through the W-β pathway [17]. Due to the species of miRNAs and the diversity of its targets, further research in this field is needed.

**Fig. 1:**
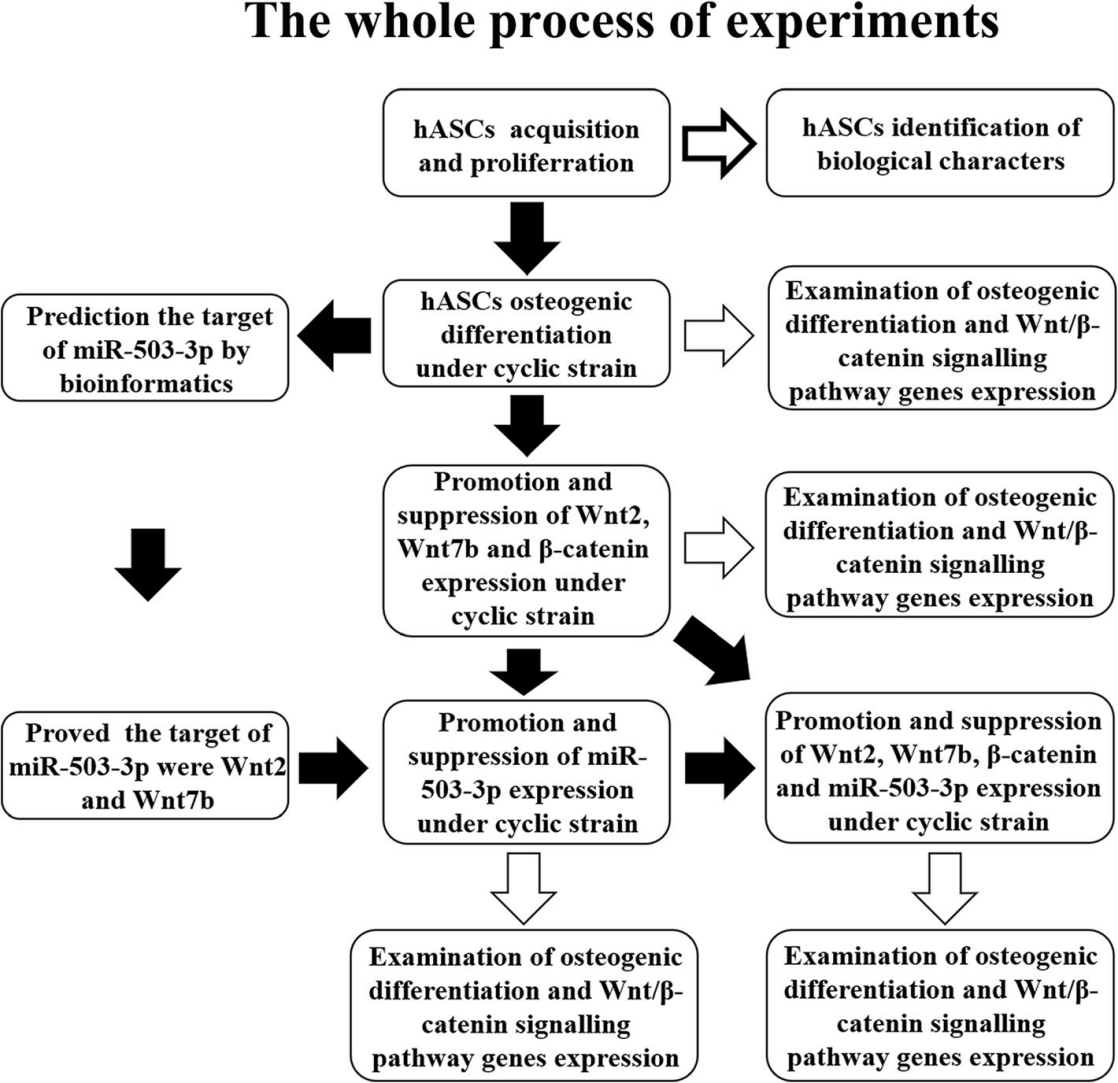
Experimental flow diagram

In prior studies carried out in our laboratory, miR-503-3p expression in hASCs was downregulated during OD in vitro under tensile strain. Our data also showed that there was activation of the W-β pathway as well [6, 18]. Based on the result of bioinformation, Wnt2 and Wnt7b, which were activators of the W-β pathway, may be targets of miR-503-3p. Therefore, we hypothesized that W-β pathway activated by Wnt2 and Wnt7b through downregulation of miR-503-3p was involved in OD of hASCs induced by cyclic strain in vitro. In this study, we tested this hypothesis by a series of experiments, and the whole process was shown in Fig. 1. The result presented herein provided a theoretical and experimental basis in promoting OD of hASCs under cyclic strain for the application of mechanical factors in bone regeneration.

## Materials and methods

### The obtain method and characterization of hASCs

The obtain method and characterization of hASCs were illustrated in our previous study [18].

### Application of cyclic strain to hASCs

The 4th passage of hASCs was plated at a density of 1.0 × 10^5^ cells/ml into BioFlex™ plates (Flexcell, USA). Alpha-modified Eagle medium (α-MEM) (Gibco, USA) was used to culture the cells along with 10% fetal bovine serum (FBS) (Gibco, USA) in BioFlex™ plates for 24 h under the condition of 37 °C and 5% CO_2_. After cells were adherent to silicone rubber in BioFlex™ plates, they were loaded on uniaxial cyclic strain (5%, 0.5 Hz, 2 h/days) for 6 days in α-MEM with 10% FBS by Flexcell® FX-5000™ Compression System (Flexcell, USA) under the condition of 37 °C and 5% CO_2_. The control group was maintained under identical culture conditions just without tension stimulation. Cells were used to detect by immunofluorescence, RT-PCR, and western blot analyses after 6-day cyclic strain loading.

### Bioinformatics analysis

The sequence of miR-503-3p among species and their predicted binding sites to 3′-UTR of Wnt2 and Wnt7b were analyzed using the following database: miRecords (http://mirecords.biolead.org/), TargetScan (http://www.targetscan.org), miRGator (http://genome.ewha.ac.kr/miRGator/miRGator.html), miRWalk (http://mirwalk.umm.uni-heidelberg.de), and miRBase (http://www.mirbase.org).

### Transfecting of tools for nucleic acid expression

The 4th passage hASCs were transfected with expression plasmid of Wnt2 (EX-Wnt2), siRNA for Wnt2 (siWnt2), expression plasmid of Wnt7b (EX-Wnt7b), siRNA for Wnt7b (siWnt7b), expression plasmid for negative control (EX-Ctrl), negative control of siRNA (siR-Ctrl), miR-503-3p mimic, negative control of miR-503-3p mimic (miR-Ctrl mimic), miR-503-3p inhibitor, negative control of miR-503-3p inhibitor (miR-Ctrl inhibitor), Wnt2 3′ UTR-Wild Type (wt-Wnt2), Wnt2 3′ UTR-Mutant (mu-Wnt2), Wnt7b 3′ UTR-Wild Type (wt-Wnt7b), and Wnt7b 3′ UTR-Mutant (mu-Wnt7b) by Lipofectamine® 2000 (Invitrogen, USA). All sequences for transfection in this section are listed in Table 1. The miR-503-3p mimic, inhibitor, siWnt2, siWnt7b, siR-Ctrl, and miR-Ctrl inhibitor and mimic, were obtained from GenePharma Corporation (Shanghai, China). The rest of mentioned tools for nucleic acid expression in this paragraph were obtained from GeneCopoeia Corporation (Guangzhou, China).

**Table 1:** The constructed sequences used in this study

Genes	Sequence	
miR-503-3p mimic		5′-GGGGUAUUGUUUCCGCUGCCAGGdTdT-3′
miR-Ctrl mimic		5′-UUGUACUACACAAAAGUACUGdTdT-3′
miR-503-3p inhibitor		5′-CCUGGCAGCGGAAACAAUACCCCdTdT-3′
miR-Ctrl inhibitor		5′-CAGUACUUUUGUGUAGUACAAdTdT-3′
siWnt2 siWnt7b	Sense	5′-GCCUUUGUUUAUGCCAUCUdTdT-3′
	Antisense	5′-AGAUGGCAUAAACAAAGGCdTdT-3′
	Sense	5′-CAGACCUGGUGUACAUUGAdTdT-3′
	Antisense	5′-UCAAUGUACACCAGGUCUGdTdT-3′
siR-Ctrl	Sense	5′-UUCUCCGAACGUGUCACGUdTdT-3′
	Antisense	5-ACGUGACACGUUCGGAGAAdTdT-3

Lentiviral particles that contain shRNA control or shRNA targeting β-catenin (Mission Lentiviral Transduction Particles from Sigma-Aldrich, USA) were transduced into SKOV3 cells to control the β-catenin expression. After transducing, puromycin (1.5 μM/mL) was used to select the SKOV3 cells. Individual siRNA sequences (#1: GCGUUUGGCUGAACCAUCA and #2: UAAUGAGGACCUAUACUUA, Dharmacon) or a pool of 4 short interfering RNAs that targeted β-catenin (siβ-catenin; Dharmacon, USA; siGenome SMART pool) were used to transfect performing by Dream FECT transfection reagent (Oz Biosciences, France). The control, scrambled siRNA pool (Dharmacon), was maintained.

### Dual luciferase reporter assay

In this assay, insertion of the synthetic fragments of wt-Wnt2 or corresponding mu-Wnt2 that contained the predicted seed match site with miR-503-3p, were carried out between the Not I and Xho I cleavage sites of the psiCHECK-2 vector (Promega, USA), downstream of the *Renilla* luciferase reporter gene. HEK-293 T cells, on seeding into a white and opaque 96-well plate at 70% confluence, were co-transfected with each reporter construct (pmirGLO-wt-Wnt2, pmirGLO-mu-Wnt2, pmirGLO-wt-Wnt7b, or pmirGLO-mu-Wnt7b) and Lv-miR-503-3p, Lv-miR-NC, Lv-ASO-503-3p, or LvASO-NC. According to the protocols of the manufacturer, Firefly and *Renilla* luciferase activity was detected 48 h after transfection with the Dual Luciferase Reporter Gene Assay Kit (Beyotime, China). This was done by normalization of firefly values *Renilla* luciferase.

### β-Catenin in hASCs detected by immunofluorescence

After transfecting process mentioned in transfecting of tools for nucleic acid expression, the effect of miR-503-3p, Wnt2, and Wnt7b on the expression of β-catenin protein and their location were detected by immunofluorescence. After loading cyclic strain, samples were fixed by 4% paraformaldehyde in wells of BioFlex™ plates, then rinsing 3 times by PBS. At room temperature, 0.5% Triton X-100 (Sigma-Aldrich, USA) was added for transpiring them for 20 min. Goat serum (Beyotime, China) was used to block hASCs for 2 h and then incubated with primary antibodies specific for β-catenin (1:1000, Abcam, USA) overnight at 4 °C. After it is rinsed thrice with PBS with Tween-20, cells were incubated with fluorescent Cy3 secondary antibodies (1:50, Proteintech, USA) for 1 h at 37 °C in the dark. Sample photos were taken by fluorescence microscopy (ZEISS, Germany).

### Detection of gene expression with RT-PCR

Total RNA isolated from cells by Trizol Reagent (Invitrogen, USA). PrimeScript RT Master Mix Perfect Real-Time (TaKaRa, Japan) was used to synthesize cDNA from RNA. ABI 7300 Real-Time PCR System was used to carry out RT-PCR reactions (Applied Biosystems, UK). RUNX2, alkaline phosphatase (ALP), secreted protein acidic and cysteine rich (SPARC), Wnt2, Wnt7b, β-catenin, and glyceraldehyde-3-phosphate dehydrogenase (GAPDH) were detected by SYBR Premix ExTaq kit (TaKaRa, Japan). Primers designing and synthesis was done by the RiboBio Corporation (Guangzhou, China), and sequences of primers listed in Table 2. The results of RT-PCR analysis were calculated by − ΔΔCt method.

**Table 2:** The sequence of primer used for RT-PCR in this study

Gene	Accession No.	5′-3′	Tm (°C)
RUNX2	NM_001015051	F: TAGATAGTGATTGCGTTTGGCTATG	60
		R: CACTAAGAAATGTTTCAAGGGTCC	60
ALP	NM_003064	F: GAAAGTCCTTCAAAGCTGGAGTCT	60
		R: TCTGGCACTCAGGTTTCTTGTATC	60
SPARC	NM_001309443	F: TGTGATCTAAATCCACTCCTTCCA	60
		R: ACAAACCATCCAAACATTTTAAACA	60
Wnt2	NM_004185	F: GGGGCACGAGTGATCTGTG	62
		R: GCATGATGTCTGGGTAACGCT	62
Wnt7b	NM_058238	F: CACAGAAACTTTCGCAAGTGG	60
		R: GTACTGGCACTCGTTGATGC	60
β-catenin	XM_024453360	F: TTGAACTGTTTGAGGCGAAGAG	60
		R: ACTGAACACCGAGTTAGAGGAAT	60
GAPDH	NM_001256799.2	F: GAACGGGAAGCTCACTGG	60
		R: GCCTGCTTCACCACCTTCT	60

### Western blot analysis for protein expression detection

Cell lysis buffer for Western and IP Kit (Beyotime, China) was used for total protein extraction; nuclear and cytoplasmic protein extraction kit was used to isolate the nuclear and cytoplasmic protein (Beyotime, China). Following centrifugation at 4 °C (12,000×*g*, 15 min), bicinchoninic acid kit was used to quantify the protein concentrations (Beyotime, China). Protein samples were separated by sodium dodecyl sulfate polyacrylamide gel electrophoresis and transferred onto membranes (GE Healthcare Life Sciences, USA). Freshly prepared Tris-buffer saline (TBS) that contained 5% non-fat milk was utilized for blocking the membranes for 2 h at room temperature. The blots were probed with primary antibodies at 4 °C overnight, in the dilution ratio 1:1000. The primary antibodies used in western blot were listed as follows: RUNX2 (Abcam, USA), ALP (Abcam, USA), SPARC (Abcam, USA), Wnt7b (R&D Systems, USA), Wnt2 (R&D Systems, USA), GAPDH (Cell Signaling Technology, USA), and β-catenin (Abcam, USA) and followed by washing of membranes thrice in TBS-0.05% Tween 20, followed by incubation at room temperature with the corresponding secondary antibodies for 1 h. Blots were then incubated in the dark with ECL (Thermo Fisher Scientific, Germany) and visualized by exposing to enhanced chemiluminescence reagents (GE Healthcare, USA). “ImageJ software 1.4.3.67” (National Institutes of Health, USA) was used to analyze the grayscales of blots.

### Detection of ALP activity and calcium with colorimetric assay

The cells were lysed by cell lysis buffer for western and IP kit (Beyotime, China) following the manual of the manufacturers. Alkaline phosphatase assay kit (Beyotime, China) was used to detect the ALP activity in cell lysates. Samples and standards were added in 96-well plates, respectively. Then, para-nitrophenyl phosphate solution and ALP enzyme solution was added in sample and standard wells respectively and incubated for 10 min at 37 °C. Stop solution utilized to terminate the reaction. The 96-well plates analyzed with a microplate spectrophotometer (SpectraMax M2e, Molecular Devices, USA) at 405 nm.

The calcium in cells was detected by calcium colorimetric assay kit (Beyotime, China). The cells were lysed by sample lysis solution in the kit. Following centrifugation at 4 °C (12,000×*g*, 5 min), the supernatant was used for detecting. Fifty microliters standard and sample were added in 96-wells plates following the manual of manufactures. Test solution (test buffer to o-cresolphthalein complexone = 1:1) was added to each well (150 μl per well) and incubated for 10 min at room temperature in the dark. The 96-well plates analyzed with a microplate spectrophotometer (SpectraMax M2e, Molecular Devices, USA) at 575 nm.

### Statistical analysis

All data and statistical analysis were done with SPSS 22.0 (IBM-Corp., USA). Comparisons between groups were analyzed using the Student’s *t* tests (two-sided) or analysis of variance for experiments with more than two subgroups. The standard of statistical significance was *p* < 0.05. All quantifiable results presented as the pattern of mean ± standard error.

## Results

### Wnt2 and Wnt7b were targets of miR-503-3p

**Fig. 2:**
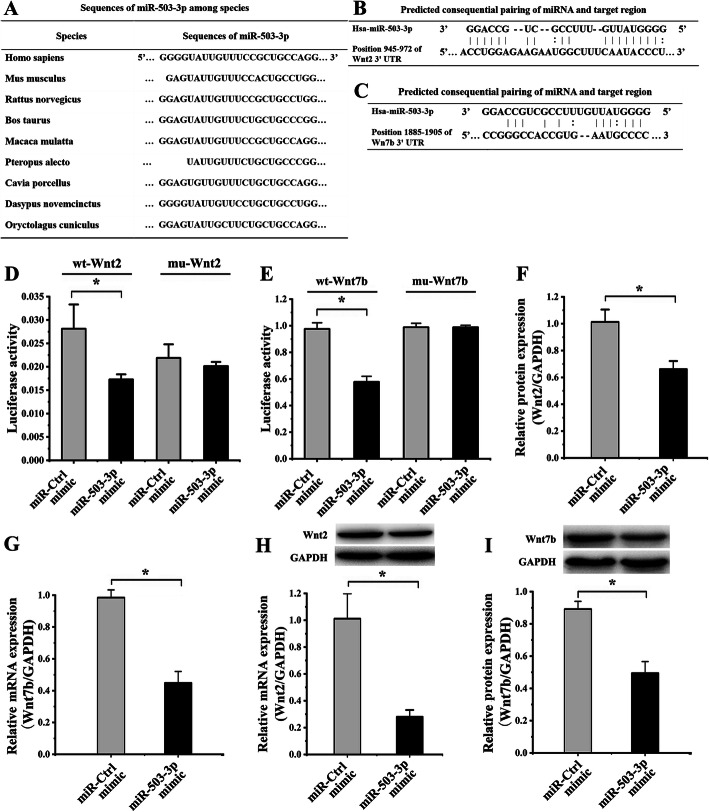
Wnt2 and Wnt7b were targets of miR-503-3p. (*p < 0.05, there were significant differences between these two groups) **a** The sequences of miR-503-3p are highly conservative across species. **b** miR-503-3p might bind to Wnt2 3′-UTR. **c** miR-503-3p might bind to Wnt7b 3′-UTR. **d** The luciferase activity of hASCs co-transfected with miR-503-3p mimic and wt-Wnt2 significantly reduced, compared to the miR-Ctrl mimic and wt-Wnt2 group. The luciferase activity of hASCs co-transfected with miR-503-3p mimic and mut-Wnt2 had no significance, compared to miR-Ctrl mimic and mut-Wnt2 group. **e** The luciferase activity of hASCs co-transfected with miR-503-3p mimic and wt-Wnt7b significantly reduced, compared to the miR-Ctrl mimic and wt-Wnt7b group. The luciferase activity of hASCs co-transfected with miR-503-3p mimic and mut-Wnt7b had no significance, compared to miR-Ctrl mimic and mut-Wnt7b group. **f** Compared to the miR-Ctrl mimic group, Wnt2 mRNA levels decreased in hASCs transfected with miR-503-3p mimic as shown by real-time PCR. **g** Compared to the miR-Ctrl mimic group, Wnt7b mRNA levels decreased in hASCs transfected with miR-503-3p mimic as shown by real-time PCR. **e** Protein blots were listed in the upper column. Compared to the miR-Ctrl mimic group, Wnt2 protein levels decreased in hASCs transfected miR-503-3p mimic as shown by Western blot. **f** Protein blots were listed in the upper column. Compared to the miR-Ctrl mimic group, Wnt7b protein levels decreased in hASCs transfected miR-503-3p mimic as shown by western blot

Based on the result of bioinformatics databases, the sequence of miR-503-3p in many species is conservative (Fig. 2a). Wnt2 3′-UTR and Wnt7b 3′-UTR could match with miR-503-3p (Fig. 2b, c).

To investigate the molecular mechanisms and determine whether miR-503-3p directly targeted Wnt2 and Wnt7b in hASCs, luciferase reporter gene assays were performed by constructed wt-Wnt2, mu-Wnt2, wt-Wnt7b, and mu-Wnt7b binding site mutagenesis of miR-503-3p. Then, above vectors and miR-503-3p mimic were co-transfected into HEK-293T cells.

The results showed that hASCs transfected with miR-503-3p mimic and wt-Wnt2 significantly reduced the luciferase activity 1.63 ± 0.39-fold (F) (*p* = 0.024) when compared with hASCs transfected with miR-Ctrl mimic (MCM) and wt-Wnt2 (Fig. 2d). Moreover, the luciferase activity of hASCs transfected with miR-503-3p mimic and mut-Wnt2 had no statistical significance, compared with hASCs transfected with MCM and mu-Wnt2 (*p* = 0.377) (Fig. 2d), indicating the inhabitation of luciferase activity regulated by miR-503-3p mimic was broken by the mut-Wnt2.

The results showed that hASCs transfected with miR-503-3p mimic containing wt-Wnt7b significantly reduced the luciferase activity 1.69 ± 0.02-F (*p* < 0.001) in comparison with hASCs that were transfected with wt-Wnt7b and MCM (Fig. 2e). Moreover, the luciferase activity of hASCs transfected with miR-503-3p mimic containing mu-Wnt7b had no statistical significance, compared with hASCs transfected with mu-Wnt7b and MCM (*p* = 1.00) (Fig. 2e), indicating the inhabitation of luciferase activity regulated by miR-503-3p mimic was broken by the mu-Wnt7b.

Compared to hASCs transfected with MCM, Wnt2 and Wnt7b mRNA levels in hASCs transfected with miR-503-3p mimic were repressed 3.60 ± 0.23-F (*p* = 0.003) and 2.32 ± 0.04-F (*p* = 0.001) (Fig. 2f, g); the western blot analysis also suggested both Wnt2 and Wnt7b protein levels were also decreased 1.53 ± 0.07-F (*p* = 0.005) and 1.80 ± 0.07-F (*p* = 0.001) (Fig. 2h, i). This shows that Wnt2 and Wnt7b were target genes mediated by miR-503-3p at both transcriptional and translational levels.

### hASCs OD induced by cyclic strain

**Fig. 3:**
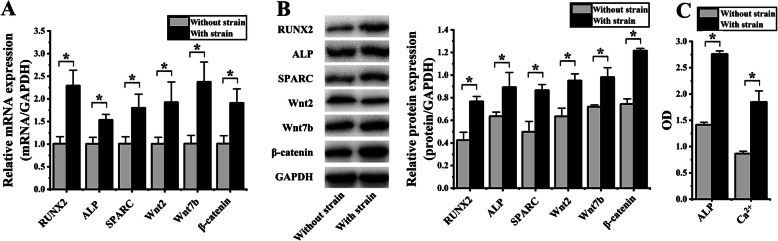
The cyclic strain induced osteogenic differentiation of hASCs. (*p < 0.05, there were significant differences between these two groups) **a** After cyclic strain loading for 6 days, the mRNA expression of RUNX2, ALP, SPARC, Wnt7b, and β-catenin were significantly increased, compared to hASCs without cyclic strain loading. **b** Protein blots were listed in the left column. After cyclic strain loading for 6 days, the protein expression of RUNX2, ALP, SPARC, Wnt7b, and β-catenin were significantly increased, compared to hASCs without cyclic strain loading. **c** After cyclic strain loading for 6 days, both the ALP activity and the content of Ca^2+^ were increased

Compared to hASCs without cyclic strain loading, the mRNA expression associated with OD (RUNX2, ALP, and SPARC; R, A, S) showed a significant increase with 2.22 ± 0.01-F (*p* = 0.002), 1.39 ± 0.01-F (*p* = 0.002), and 1.62 ± 0.06-F (*p* = 0.002), respectively; the W-β pathway genes (Wnt2, Wnt7b, and β-catenin; W2, W7, β) showed an increase of 1.91 ± 0.17-F (*p* = 0.028), 1.72 ± 0.05-F (*p* = 0.034), and 1.47 ± 0.11-F (*p* = 0.014), respectively (Fig. 3a).

In comparison to hASCs without cyclic strain loading, the R, A, S protein expression exhibited a significant increase 1.81 ± 0.28-F (*p* = 0.002), 1.40 ± 0.14-F (*p* = 0.030), and 1.74 ± 0.22-F (*p* = 0.004), respectively; W2, W7, β showed an increase of 1.50 ± 0.14-F (*p* = 0.004), 1.36 ± 0.11-F (*p* = 0.006), and 1.63 ± 0.08-F (*p* < 0.001), respectively (Fig. 3b).

Compared to hASCs without cyclic strain loading, the ALP activity showed a significant increase with 1.95 ± 0.05-F (*p* < 0.001); the content of Ca^2+^ showed an increase of 2.13 ± 0.17-F (*p* = 0.001) (Fig. 3c).

### Role of Wnt2 on hASCs osteogenic differentiation induced by cyclic strain

**Fig. 4:**
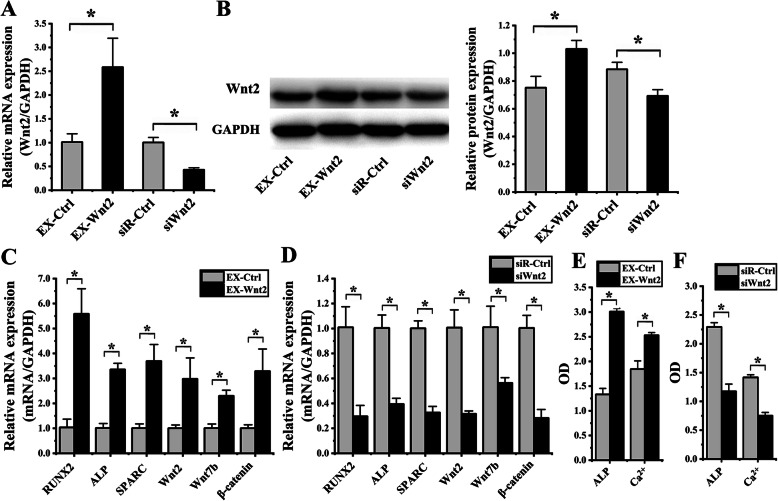
Effect of Wnt2 on osteogenic differentiation of hASCs under cyclic strain. (*p < 0.05, there were significant differences between these two groups) **a** The results of real-time PCR showed that the Wnt2b expression in EX-Wnt2b group increased compared to the EX-Ctrl group; Wnt2 expression in the siWnt2 group significantly decreased, compared to the siR-Ctrl group. **b** Protein blots were listed in the left column. The results of western blot showed that Wnt2 expression in the EX-Wnt2 group increased, compared to the EX-Ctrl group; Wnt2 expression in the siWnt2 group significantly decreased, compared to the siR-Ctrl group. **c** After EX-Wnt2 transfection, the results of real-time PCR showed that RUNX2, ALP, SPARC, Wnt7b, and β-catenin significantly enhanced, compared to the EX-Ctrl group. **d** After siWnt2 transfection, the results of real-time PCR showed that RUNX2, ALP, SPARC, Wnt7b, and β-catenin significantly decreased, compared to the siR-Ctrl group. **e** Comparing to the EX-Ctrl group, both the ALP activity and the content of Ca^2+^ were significantly increased when EX-Wnt2 transfected. **f** Comparing to the siR-Ctrl group, both the ALP activity and the content of Ca^2+^ were significantly decreased when siWnt2 transfected

For determining effects of transfection, Wnt2 expressions of hASCs, which were transfected with the EX-Wnt2, siWnt2, EX-Ctrl, and siR-Ctrl, were examined by immunofluorescence, real-time PCR, and western blot. The results of RT-PCR analysis showed that the Wnt2 expression in EX-Wnt2 group increased 2.56 ± 0.16-F (*p =* 0.013) in comparison with EX-Ctrl group; Wnt2 expression in the siWnt2 group showed a significant decreased 2.34 ± 0.45-F (*p =* 0.001) in comparison with the siR-Ctrl group (Fig. 4a). The results of western blot analysis showed that Wnt2 expression in the EX-Wnt2 group showed an increase of 1.37 ± 0.07-F (*p* = 0.009) in comparison with the EX-Ctrl group; Wnt2 expression in the siWnt2 group showed a significant decrease 1.28 ± 0.03-F (*p* = 0.008) in comparison with the siR-Ctrl group (Fig. 4b).

Furthermore, RT-PCR analysis was used to determine effects of Wnt2 on the OD of hASCs after loading cyclic strain for 6 days. After EX-Wnt2 transfection, the results showed that R, A, S, W2, W7, β significantly enhanced 5.39 ± 2.14-F (*p* = 0.002), 3.32 ± 0.80-F (*p* < 0.001), 3.66 ± 1.20-F (*p* = 0.003), 2.96 ± 0.44-F (*p =* 0.016), 2.28 ± 0.62-F (*p* = 0.001), and 3.27 ± 0.49-F (*p* = 0.012), respectively, in comparison with the EX-Ctrl group (Fig. 4c). After siWnt2 transfected, the results showed that R, A, S, W2, W7, β significantly reduced 3.41 ± 0.09-F (*p* = 0.003), 2.55 ± 0.03-F (*p* = 0.001), 3.07 ± 0.07-F (*p <* 0.001), 3.19 ± 0.07-F (*p =* 0.001), 1.79 ± 0.11-F (*p =* 0.011), and 3.55 ± 0.10-F (*p =* 0.001) respectively, in comparison with the siR-Ctrl group (Fig. 4d). After EX-Wnt2 transfection, ALP activity and the content of Ca^2+^ significantly increased 2.26 ± 0.15-F (*p* < 0.001) and 1.37 ± 0.17-F (*p* = 0.003), respectively, in comparison with the EX-Ctrl group (Fig. 4e). After siWnt2 transfected, ALP activity and the content of Ca^2+^ significantly decreased 1.95 ± 0.19-F (*p* < 0.001) and 1.87 ± 0.11-F (*p* < 0.001), respectively, in comparison with the siR-Ctrl group (Fig. 4f).

All these results above suggested that overexpression of Wnt2 enhanced OD of hASCs, and knockdown of Wnt2 inhibited OD of hASCs.

### Role of Wnt7b on hASCs OD induced by cyclic strain

**Fig. 5:**
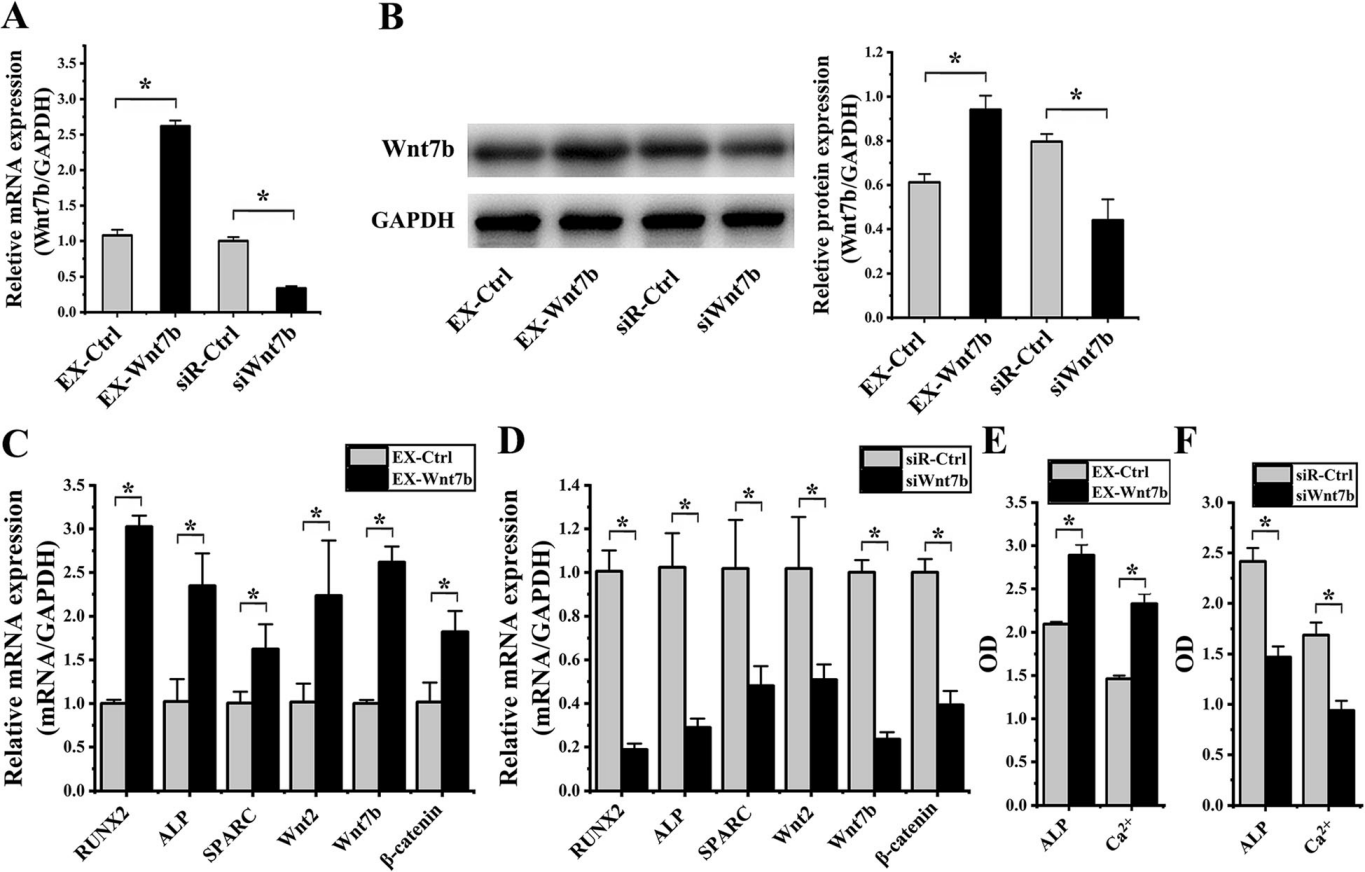
Effect of Wnt7b on osteogenic differentiation of hASCs under cyclic strain. (*p < 0.05, there were significant differences between these two groups) **a** The results of real-time PCR showed that the Wnt7b expression in EX-Wnt7b group increased, compared to the EX-Ctrl group; Wnt7b expression in the siWnt7b group significantly decreased, compared to the siR-Ctrl group. **b** Protein blots were listed in the left column. The results of western blot showed that Wnt7b expression in the EX-Wnt7b group increased, compared to the EX-Ctrl group; Wnt7b expression in the siWnt7b group significantly decreased, compared to the siR-Ctrl group. **c** After EX-Wnt7b transfection, the results of real-time PCR showed that RUNX2, ALP, SPARC, Wnt7b, and β-catenin significantly enhanced, compared to the EX-Ctrl group. **d** After siWnt7b transfection, the results of real-time PCR showed that RUNX2, ALP, SPARC, Wnt2, Wnt7b, and β-catenin significantly decreased, compared to the siR-Ctrl group. **e** Comparing to the EX-Ctrl group, ALP activity and the content of Ca^2+^ significantly increased when EX-Wnt7b transfected. Comparing to the siR-Ctrl group, ALP activity and the content of Ca^2+^ significantly decreased when siWnt7b transfected

For determining effects of transfection, Wnt7b expressions of hASCs, which were transfected with the EX-Wnt7b, siR-Ctrl, EX-Ctrl, and siWnt7b were examined by real-time PCR, immunofluorescence, and western blot. The results of RT-PCR analysis indicated that the Wnt7b expression in EX-Wnt7b group increased 2.43 ± 0.10-F (*p* < 0.001) in comparison with the EX-Ctrl group; Wnt7b expression in the siWnt7b group showed a significant decrease 2.98 ± 0.02-F (*p* < 0.001) in comparison with the siR-Ctrl group (Fig. 5a). The results of western blot analysis showed that Wnt7b expression in the EX-Wnt7b group increased 1.54 ± 0.12-F (*p* = 0.003) in comparison with the EX-Ctrl group; Wnt7b expression in the siWnt7b group showed a significant reduction 1.81 ± 0.10-F (*p* = 0.003) in comparison with the siR-Ctrl group (Fig. 5b).

Furthermore, RT-PCR analysis was used to determine effects of Wnt7b on the OD of hASCs after loading cyclic strain for 6 days. After EX-Wnt7b transfection, the results showed that R, A, S, W2, W7, β significantly enhanced 3.03 ± 0.01-F (*p* < 0.001), 2.29 ± 0.22-F (*p* = 0.007), 1.61 ± 0.10-F (*p* = 0.027), 2.20 ± 0.38-F (*p =* 0.034), 2.62 ± 0.12-F (*p <* 0.001), and 1.79 ± 0.17-F (*p =* 0.013), respectively, compared to the EX-Ctrl group (Fig. 5c). After siWnt7b transfected, the results showed that R, A, S, W2, W7, β significantly decreased 5.33 ± 0.01-F (*p* < 0.001), 3.53 ± 0.02-F (*p* = 0.001), 2.12 ± 0.03-F (*p* = 0.018), 2.00 ± 0.54-F (*p =* 0.023), 4.24 ± 0.40-F (*p <* 0.000), and 2.55 ± 0.27-F (*p <* 0.001), respectively, compared to the siR-Ctrl group (Fig. 5d). After EX-Wnt7b transfection, ALP activity and the content of Ca^2+^ significantly increased 1.38 ± 0.08-F (*p* = 0.008) and 1.59 ± 0.04-F (*p* < 0.001), respectively, compared to the EX-Ctrl group (Fig. 5e). After was siWnt7b transfected, ALP activity and the content of Ca2+ significantly decreased 1.65 ± 0.16-F (*p* = 0.001) and 1.80 ± 0.15-F (*p* = 0.001), respectively, compared to the siR-Ctrl group (Fig. 5f).

All these results above suggested that overexpression of Wnt7b enhanced OD of hASCs, and knockdown of Wnt7b inhibited OD of hASCs.

### Role of β-catenin on hASCs OD induced by cyclic strain

**Fig. 6:**
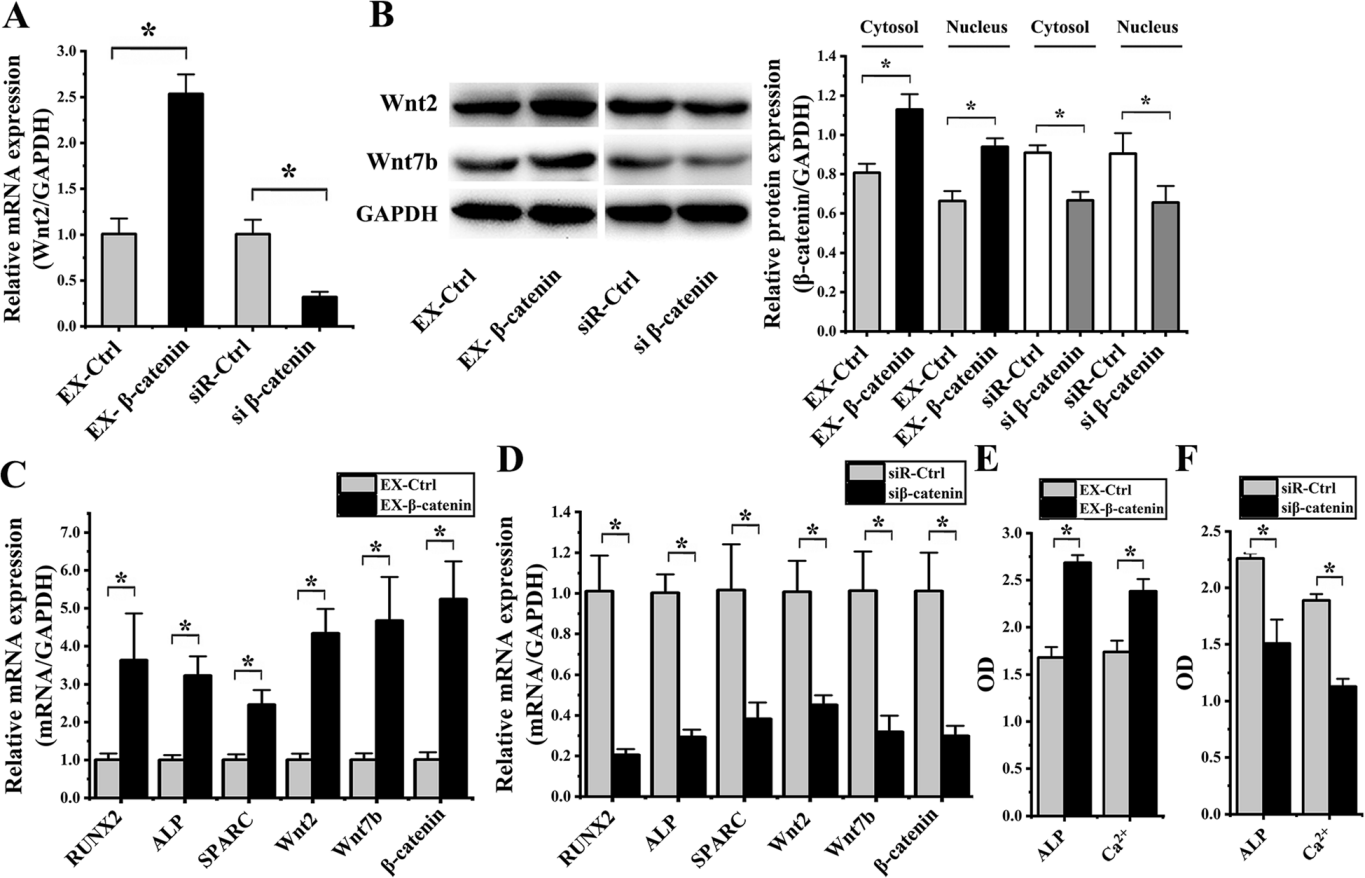
Effect of β-catenin on osteogenic differentiation of hASCs under cyclic strain. (*p < 0.05, there were significant differences between these two groups) **a** The results of real-time PCR showed that the β-catenin expression in EX-β-catenin group increased, compared to the EX-Ctrl group; β-catenin expression in the siβ-catenin group significantly decreased, compared to the siR-Ctrl group. **b** Protein blots were listed in the left column. The results of western blot showed that cytoplasmic β-catenin and nuclear β-catenin expression in the EX-β-catenin group increased, compared to the EX-Ctrl group; cytoplasmic β-catenin and nuclear β-catenin expression in the siβ-catenin group significantly decreased, compared to the siR-Ctrl group. **c** After EX-β-catenin transfection, the results of real-time PCR showed that RUNX2, ALP, SPARC, Wnt2, Wnt7b, and β-catenin significantly enhanced, compared to the EX-Ctrl group. **d** After siβ-catenin transfection, the results of real-time PCR showed that RUNX2, ALP, SPARC, Wnt2, Wnt7b, and β-catenin significantly decreased, compared to the siR-Ctrl group. **e** Comparing to the EX-Ctrl group, ALP activity and the content of Ca^2+^ significantly increased when EX-β-catenin transfected. Comparing to the siR-Ctrl group, ALP activity and the content of Ca^2+^ significantly decreased when siβ-catenin transfected

For determining effects of transfection, β-catenin expressions of hASCs, which were transfected with the EX-β-catenin, siβ-catenin, EX-Ctrl, and siR-Ctrl, were examined by RT-PCR, western blotting, and immunofluorescence. The results of RT-PCR analysis indicated that the β-catenin expression in EX-β-catenin group showed an increase of 2.51 ± 0.69-F (*p* = 0.001) in comparison to the EX-Ctrl group; the β-catenin expression in the siβ-catenin group significantly reduced 3.15 ± 0.89-F (*p* = 0.002) in comparison to the siR-Ctrl group (Fig. 6a). The results of western blot analysis indicated that the cytoplasmic and nuclear β-catenin expression in the EX-β-catenin group showed an increase of 1.40 ± 0.07-F (*p* = 0.004) and 1.42 ± 0.04-F (*p* = 0.002) in comparison to the EX-Ctrl group; the cytoplasmic β-catenin and nuclear β-catenin expression in the siβ-catenin group significantly reduced 1.38 ± 0.12-F (*p* = 0.002) and 1.38 ± 0.09-F (*p* = 0.032) in comparison to the siR-Ctrl group (Fig. 6b).

Furthermore, RT-PCR analysis was used to determine effects of β-catenin on the OD of hASCs after loading cyclic strain for 6 days. After EX-β-catenin transfection, the results showed that R, A, S, W2, W7, β significantly enhanced 3.60 ± 1.07-F (*p* = 0.022), 3.21 ± 0.49-F (*p* = 0.002), 2.45 ± 0.29-F (*p* = 0.004), 4.30 ± 1.12-F (*p* = 0.001), 4.63 ± 1.88-F (*p* = 0.006), and 5.17 ± 1.04-F (*p* = 0.002), respectively, in comparison to the EX-Ctrl group (Fig. 6c). After siβ-catenin transfected, the results showed that R, A, S, W2, W7, β significantly decreased 4.92 ± 1.46-F (*p* = 0.001), 3.41 ± 0.38-F (*p* < 0.001), 2.66 ± 1.27-F (*p* = 0.010), 2.23 ± 0.33-F (*p* = 0.004), 3.17 ± 1.27-F (*p* = 0.005) and 3.38 ± 0.10-F (*p* = 0.003) respectively, compared to the siR-Ctrl group (Fig. 6d). After EX-β-catenin transfection, ALP activity and the content of Ca^2+^ significantly increased 1.60 ± 0.16-F (*p* < 0.001) and 1.37 ± 0.12-F (*p* = 0.003), respectively, in comparison to the EX-Ctrl group (Fig. 6e). After siβ-catenin transfected, ALP activity and the content of Ca2+ significantly decreased 1.50 ± 0.19-F (*p* = 0.021) and 1.67 ± 0.06-F (*p* < 0.001), respectively, compared to the siR-Ctrl group (Fig. 6f).

All these results above suggested that overexpression of β-catenin enhanced OD of hASCs, and knockdown of β-catenin inhibited OD of hASCs.

### Role of miR-503-3p overexpression and inhibition on hASCs OD induced by cyclic strain

**Fig. 7:**
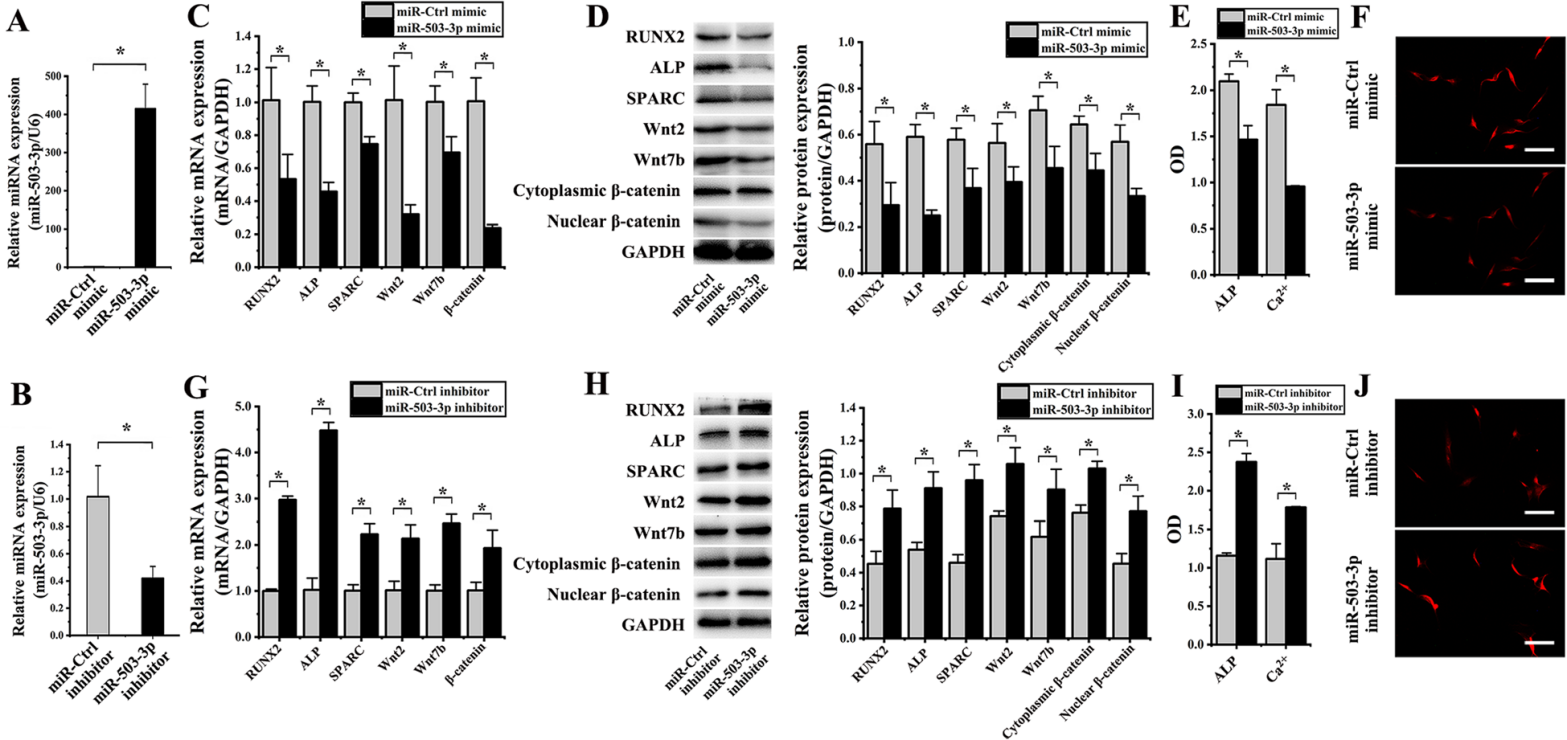
Effects of miR-503-3p overexpression and inhibition on osteogenic differentiation of hASCs under cyclic strain. (*p < 0.05, there were significant differences between these two groups) **a** The expression of miR-503-3p in hASCs transfected with miR-503-3p mimic markedly increased, compared to the miR-Ctrl mimic group. **b** The expression of miR-503-3p in hASCs transfected with miR-503-3p inhibitor decreased, compared to the miR-Ctrl inhibitor group. **c** After miR-503-3p mimic transfection, the results of real-time PCR showed that RUNX2, ALP, SPARC, Wnt2, Wnt7b, and β-catenin decreased, compared to the miR-Ctrl mimic group. **d** Protein blots were listed in the left column. After miR-503-3p mimic transfection, the results of western blot showed that RUNX2, ALP, SPARC, Wnt2, Wnt7b, cytoplasmic β-catenin, and nuclear β-catenin decreased. **e** Both ALP activity and the content of Ca^2+^ significantly decreased when miR-503-3p mimic transfected. **f** After miR-503-3p mimic transfection, the immunofluorescence intensity of β-catenin in the hASC nucleus was decreased. **g** After miR-503-3p inhibitor transfection, the results of real-time PCR showed that RUNX2, ALP, SPARC, Wnt2, Wnt7b, and β-catenin increased, compared to the miR-Ctrl inhibitor group. **h** Protein blots were listed in the left column. After miR-503-3p inhibitor transfection, the results of western blot showed that RUNX2, ALP, SPARC, Wnt2, Wnt7b, cytoplasmic β-catenin, and nuclear β-catenin decreased, compared to the miR-Ctrl inhibitor group. **i** Both ALP activity and the content of Ca^2+^ significantly increased when miR-503-3p inhibitor transfected. **j** After miR-503-3p inhibitor transfection, the immunofluorescence intensity of β-catenin in the hASC nucleus was increased

For determining efficiency of transfection with miR-503-3p mimic and inhibitor, miR-503-3p expressions in hASCs were detected by RT-PCR and western blot. After transfecting miR-503-3p mimic in hASCs, miR-503-3p markedly increased 411.49 ± 82.72-F (*p* < 0.001) than the group of MCM (Fig. 7a). After transfecting miR-503-3p inhibitor in hASCs, the expression of miR-503-3p reduced 2.43 ± 0.02-F (*p* = 0.006) than the group of miR-Ctrl inhibitor (Fig. 7b).

Furthermore, the role of miR-503-3p on hASCs OD was determined by RT-PCR, western blotting, colorimetric assay, and immunofluorescence. Compared to the MCM group, RT-PCR analysis was used to detect mRNA expression levels in hASCs transfected with miR-503-3p mimic, results indicated that R, A, S, W2, W7, β showed a decrease of 1.90 ± 0.15-F (*p* = 0.028), 2.20 ± 0.02-F (*p* = 0.001),1.34 ± 0.08-F (*p* = 0.004), 3.17 ± 1.10-F (*p* = 0.005), 1.44 ± 0.20-F (*p* = 0.018), and 4.23 ± 0.50-F (*p* = 0.001), respectively (Fig. 7c); proteins of R, A, S, W2, W7, β cytoplasmic and nuclear detected by western blot reduced 1.90 ± 0.97-F (*p* = 0.030), 2.37 ± 0.38-F (*p* = 0.001), 1.57 ± 0.24-F (*p* = 0.022), 1.43 ± 0.15-F (*p* = 0.038), 1.55 ± 0.16-F (*p* = 0.007), 1.45 ± 0.25-F (*p* = 0.014), and 1.71 ± 0.26-F (*p* = 0.007), respectively (Fig. 7d); ALP activity and the content of Ca^2+^ significantly decreased 1.43 ± 0.14-F (*p* = 0.003) and 1.93 ± 0.16-F (*p* = 0.011), respectively (Fig. 7e); β-catenin expression in cytoplasmic and nucleus showed a decrease in hASCs transfected with the miR-503-3p mimic detected by immunofluorescence (Fig. 7f).

Compared to the miR-Ctrl inhibitor group, RT-PCR analysis was used to detect the following mRNA expression levels in hASCs that had undergone transfection with miR-503-3p inhibitor, results indicated that R, A, S, W2, W7, β showed an increase of 2.97 ± 0.11-F (*p* < 0.001), 4.38 ± 1.17-F (*p* < 0.001), 2.22 ± 0.53-F (*p* = 0.001), 2.10 ± 0.44-F (*p* = 0.006), 2.45 ± 0.30-F (*p* < 0.001), and 1.91 ± 0.24-F (*p* = 0.021) respectively (Fig. 7g); the protein expression of above markers detected by western blot reduced 1.73 ± 0.13-F (*p* = 0.023), 1.69 ± 0.04-F (*p* = 0.004), 2.09 ± 0.09-F (*p* = 0.001), 1.43 ± 0.07-F (*p* = 0.006), 1.46 ± 0.34-F (*p* = 0.009), 1.35 ± 0.06-F (*p* = 0.002), and 1.70 ± 0.29-F (*p* = 0.008), respectively (Fig. 7h); ALP activity and the content of Ca^2+^ significantly increased 2.05 ± 0.05-F (*p* < 0.001) and 1.60 ± 0.30-F (*p* = 0.004), respectively (Fig. 7i); β-catenin expression in cytoplasmic and nucleus were increased in hASCs transfected with miR-503-3p mimic detected by immunofluorescence (Fig. 7j).

Our findings suggested that the overexpression of miR-503-3p inhibited OD of hASCs, while the suppression of miR-503-3p promoted its OD of hASCs.

### Roles of Wnt2 and Wnt7b regulated by miR-503-3p on hASCs OD induced by cyclic strain

Real-time PCR, western blotting, colorimetric assay, and immunofluorescence were utilized to investigate the molecular mechanisms of miR-503-3p on OD through regulating Wnt2 and Wnt7b expression in hASCs induced by cyclic strain.

**Fig. 8:**
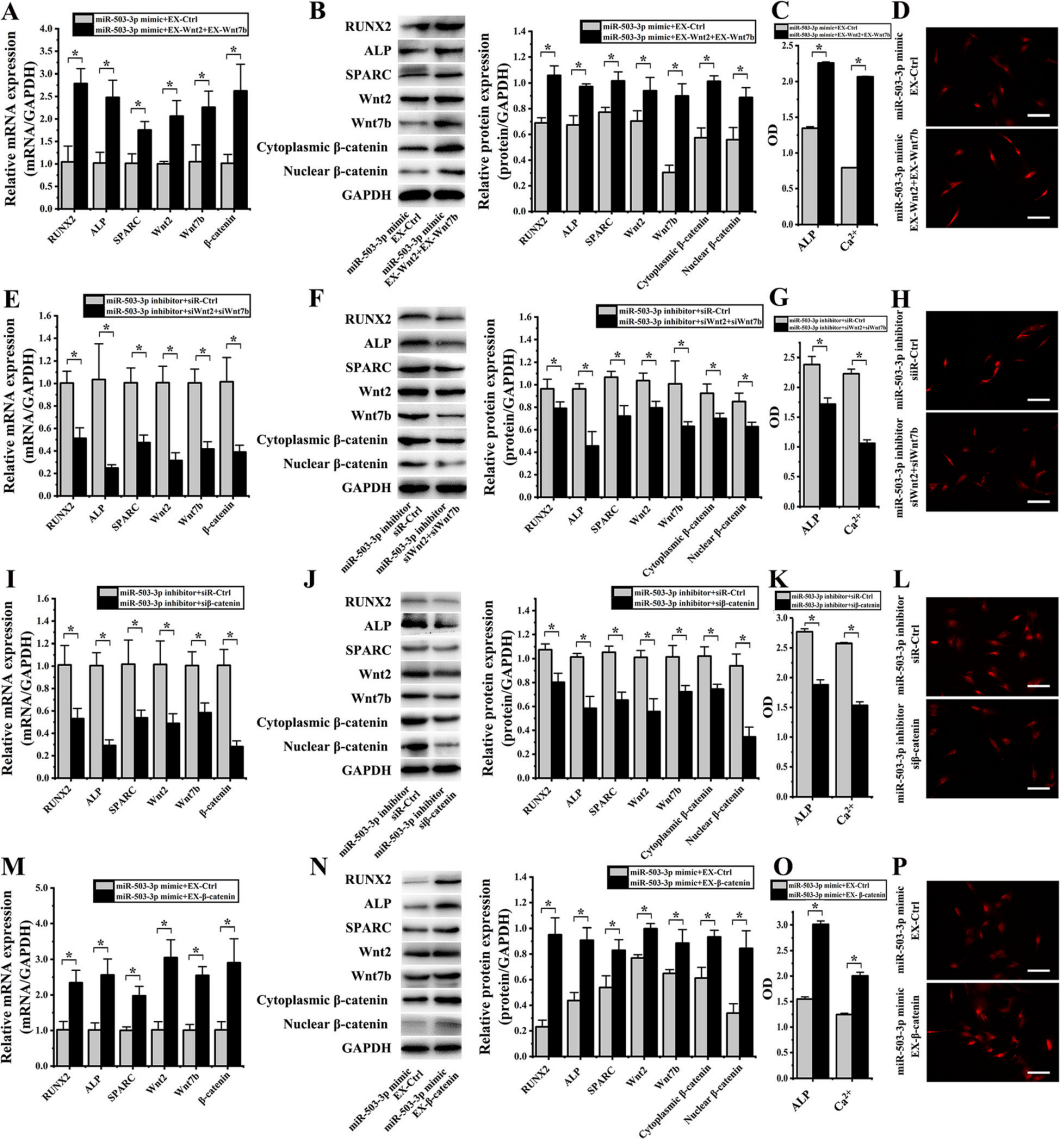
Effect of Wnt2 and Wnt7b regulated by miR-503-3p on osteogenic differentiation of hASCs under cyclic strain (*p < 0.05, there were significant differences between these two groups) **a** After co-transfecting miR-503-3p mimic with EX-Wnt2 and EX-Wnt7b, real-time PCR showed RUNX2, ALP, SPARC, Wnt2, Wnt7b, and β-catenin were increased, than the miR-503-3p mimic and EX-Ctrl group. **b** Protein blots were listed in the left column. After co-transfecting miR-503-3p mimic with EX-Wnt2 and EX-Wnt7b, western blot showed that RUNX2, ALP, SPARC, Wnt2, Wnt7b, cytoplasmic, and nuclear β-catenin increased, than the miR-503-3p mimic and EX-Ctrl group. **c** Both ALP activity and the content of Ca^2+^ significantly increased when co-transfecting miR-503-3p mimic with EX-Wnt2 and EX-Wnt7b. **d** After co-transfecting miR-503-3p mimic with EX-Wnt2 and EX-Wnt7b, the immunofluorescence intensity of nucleus β-catenin was enhanced. **e** After co-transfecting miR-503-3p inhibitor with siWnt2 and siWnt7b, real-time PCR showed that RUNX2, ALP, SPARC, Wnt2, Wnt7b, and β-catenin decreased, than the miR-503-3p inhibitor and siR-Ctrl group. **f** Protein blots were listed in the left column. After co-transfecting miR-503-3p inhibitor with siWnt2 and siWnt7b, western blot showed RUNX2, ALP, SPARC, Wnt2, Wnt7b, cytoplasmic, and nuclear β-catenin decreased, than the miR-503-3p inhibitor and siR-Ctrl group. **g** Both ALP activity and the content of Ca^2+^ significantly decreased when co-transfecting miR-503-3p inhibitor with siWnt2 and siWnt7b. **h** After co-transfecting miR-503-3p inhibitor with siWnt2 and siWnt7b, the immunofluorescence intensity of nucleus β-catenin was decreased. **i** After co-transfecting miR-503-3p inhibitor with siβ-catenin, real-time PCR showed that RUNX2, ALP, and SPARC decreased; Wnt2, Wnt7b and β-catenin decreased, than the miR-503-3p inhibitor and siR-Ctrl group. **j** Protein blots were listed in the left column. After co-transfecting miR-503-3p inhibitor with siβ-catenin, western blot showed that RUNX2, ALP, and SPARC decreased; Wnt2, Wnt7b, cytoplasmic and nuclear β-catenin decreased, than the miR-503-3p inhibitor and siR-Ctrl group. **k** Both ALP activity and the content of Ca^2+^ significantly decreased when co-transfecting miR-503-3p inhibitor with siβ-catenin. **l** After co-transfecting miR-503-3p inhibitor with siβ-catenin into hASCs, the immunofluorescence intensity of nucleus β-catenin was decreased. **m** After co-transfecting miR-503-3p mimic with EX-β-catenin, real-time PCR showed that RUNX2, ALP, and SPARC increased; Wnt2, Wnt7b, and β-catenin increased, than the miR-503-3p mimic and EX-Ctrl group. **n** Protein blots were listed in the left column. After co-transfecting miR-503-3p mimic with EX-β-catenin, western blot showed that RUNX2, ALP, and SPARC increased; Wnt2, Wnt7b, cytoplasmic, and nuclear β-catenin increased, than the miR-503-3p mimic and EX-Ctrl group. **o** Both ALP activity and the content of Ca^2+^ significantly increased when co-transfecting miR-503-3p mimic with EX-β-catenin. **p** After co-transfecting miR-503-3p mimic with EX-β-catenin, the immunofluorescence intensity of nucleus β-catenin was increased

After co-transfecting miR-503-3p mimic with EX-Wnt2 and EX-Wnt7b into hASCs, the following mRNA expression levels were detected by RT-PCR analysis, results showed that R, A, S, W2, W7, β showed an increase of 2.67 ± 1.28-F (*p* = 0.003), 2.43 ± 0.35-F (*p* = 0.005), 1.73 ± 0.19-F (*p* = 0.010), 2.06 ± 0.30-F (*p* = 0.006), 2.15 ± 1.33-F (*p =* 0.016), and 2.59 ± 0.30-F (*p* = 0.011), respectively, than the group of miR-503-3p mimic and EX-Ctrl (Fig. 8a); protein expression of R, A, S, W2, W7, β cytoplasmic and nuclear also showed an increase of 1.54 ± 0.11-F (*p =* 0.001), 1.44 ± 0.14-F (*p* = 0.002), 1.32 ± 0.03-F (*p* = 0.006), 1.34 ± 0.04-F (*p* = 0.036), 2.96 ± 0.35-F (*p* = 0.001), 1.77 ± 0.22-F (*p* = 0.001), and 1.59 ± 0.14-F (*p* = 0.010), respectively, than the group of miR-503-3p mimic and EX-Ctrl (Fig. 8b); ALP activity and the content of Ca^2+^ significantly increased 1.68 ± 0.04-F (*p* < 0.001) and 2.61 ± 0.01-F (*p* < 0.001), respectively (Fig. 8c); β-catenin expression in cytoplasmic and nucleus were increased in hASCs that had undergone transfection with the EX-Wnt2 and EX-Wnt7b detected by immunofluorescence (Fig. 8d). The above results suggested that Wnt2 and Wnt7b overexpression could remedy the inhibiting effect of miR-503-3p mimic on hASCs OD induced by cyclic strain.

After co-transfecting miR-503-3p inhibitor with siWnt2 and siWnt7b into hASCs, the following mRNA expression levels were detected by RT-PCR analysis, results showed that R, A, S, W2, W7, β reduced 1.96 ± 0.49-F (*p =* 0.004), 4.17 ± 1.79-F (*p* = 0.013), 2.12 ± 0.52-F (*p* = 0.003), 3.20 ± 1.22-F (*p =* 0.002), 2.41 ± 0.23-F (*p =* 0.002), and 2.60 ± 1.03-F (*p =* 0.008), respectively, than the group of miR-503-3p inhibitor and siR-Ctrl (Fig. 8e); proteins of R, A, S, W2, W7, β cytoplasmic and nuclear β-catenin also showed a decrease of 1.22 ± 0.15-F (*p* = 0.044), 2.12 ± 0.58-F (*p =* 0.003), 1.48 ± 0.15-F (*p =* 0.005), 1.31 ± 0.05-F (*p* = 0.009), 1.60 ± 0.23-F (*p* = 0.035), 1.32 ± 0.07-F (*p* = 0.015), and 1.36 ± 0.12-F (*p* = 0.010), respectively, than the group of miR-503-3p inhibitor and siR-Ctrl (Fig. 8f); ALP activity and the content of Ca^2+^ significantly decreased 1.39 ± 0.16-F (*p* = 0.002) and 2.10 ± 0.09-F (*p* < 0.001), respectively (Fig. 8g); the expression of β-catenin in cytoplasmic and nucleus were decreased in hASCs transfected with siWnt2 and siWnt7b detected by immunofluorescence (Fig. 8h). The above results suggested that Wnt2 and Wnt7b downregulation could weaken the potentiation of miR-503-3p inhibitor on OD of hASCs under cyclic strain.

After co-transfecting miR-503-3p inhibitor with siβ-catenin into hASCs, the following mRNA expression levels were detected by RT-PCR analysis, results indicated that R, A, S decreased 1.90 ± 0.60-F (*p* = 0.013), 3.45 ± 0.90-F (*p* = 0.001), and 1.89 ± 0.56-F (*p* = 0.021) respectively, and W2, W7, β decreased 2.08 ± 0.70-F (*p* = 0.015), 1.72 ± 0.16-F (*p* = 0.008), and 3.58 ± 0.47-F (*p* = 0.001) respectively, than the group of miR-503-3p inhibitor and siR-Ctrl (Fig. 8i); proteins of R, A, S decreased 1.34 ± 0.17-F (*p* = 0.005), 1.73 ± 0.39-F (*p* = 0.003), and 1.61 ± 0.37-F (*p* = 0.014), respectively, proteins of Wnt2, Wnt7b, cytoplasmic and nuclear β-catenin showed a decrease of 1.81 ± 0.31-F (*p* = 0.002), 1.40 ± 0.09-F (*p* = 0.001), 1.37 ± 0.15-F (*p* = 0.005), and 2.72 ± 0.55-F (*p* = 0.001), respectively, than the group of miR-503-3p inhibitor and siR-Ctrl (Fig. 8j); ALP activity and the content of Ca^2+^ significantly decreased 1.94 ± 0.02-F (*p* < 0.001) and 1.61 ± 0.06-F (*p* < 0.001), respectively (Fig. 8k); the expression of β-catenin in cytoplasmic and nucleus were decreased in hASCs co-transfected miR-503-3p inhibitor with siβ-catenin detected by immunofluorescence (Fig. 8l).

After co-transfecting miR-503-3p mimic with EX-β-catenin into hASCs, mRNA expression levels were detected by RT-PCR analysis. Results showed that R, A, S showed an increase of 2.30 ± 0.97-F (*p* = 0.006), 2.52 ± 0.40-F (*p* = 0.006), and 1.97 ± 0.46-F (*p* = 0.004) respectively, and W2, W7, β increased 3.00 ± 0.21-F (*p* = 0.003), 2.53 ± 0.26-F (*p* = 0.001), and 2.86 ± 0.82-F (*p* = 0.010), respectively, than the group of miR-503-3p mimic and EX-Ctrl (Fig. 8m); proteins of R, A, S also increased 4.10 ± 1.05-F (*p* = 0.001), 2.08 ± 0.33-F (*p* = 0.002), and 1.54 ± 0.08-F (*p* = 0.032), respectively, proteins of Wnt2, Wnt7b, cytoplasmic and nuclear β-catenin showed an increase of 1.30 ± 0.03-F (*p* = 0.001), 1.36 ± 0.12-F (*p* = 0.022), 1.52 ± 0.12-F (*p* = 0.007), and 2.49 ± 0.20-F (*p* = 0.015), respectively, than the group of miR-503-3p mimic and EX-Ctrl (Fig. 8n); ALP activity and the content of Ca^2+^ significantly increased 1.47 ± 0.04-F (*p* < 0.001) and 1.68 ± 0.02-F (*p* = 0.001), respectively (Fig. 8o); the expression of β-catenin in cytoplasmic and nucleus were increased in hASCs that had undergone co-transfection with the miR-503-3p mimic and EX-β-catenin detected by immunofluorescence (Fig. 8p).

## Discussion

Although there are many advantages of hASCs mentioned in the “Introduction” section for construction of tissue engineering, the osteogenic ability of ASCs is lower than bone mesenchymal stem cells (BMSCs). Shafiee et al. claimed that ASCs had reduced ALP activity and mineralization when compared to BMSCs during OD on the 7th and 14th day [19]. The potent osteogenic capacity of BMSCs in comparison to ASCs was also proved by Vishnubalaji et al. by the following experiments: calcium mineralization, cytochemical qualitative analysis, and RT-PCR of osteocalcin, ALP, and osteopontin [20]. Park et al. concluded the human mesenchymal stem cells were more sensitive to mechanical stimulation and more effective toward OD than the hASCs under these modes of mechanical stimulation [21]. Even so, hASCs is still one of the seed cells that can be chosen for construction of tissue engineering bone.

All tissues survive in a mechanical environment, which plays an essential role in maintaining biological activities [22–24]. In stem cells, common biomechanical stimuli that induce osteogenesis are tension, compression, and fluid shear stress. Under 5% and below strain, ALP activity and RUNX2 gene expression was found to increase in mouse bone marrow stromal cells but, on the other hand, showed a reduction with higher strains [25]. This result showed that OD is promoted by low tension levels while high levels of tension inhibit that. Compression has a role in chondrogenic as well as OD in human BMSCs. Jagodzinski et al. claimed that mesenchymal stem cells were applied to 10% cyclic compression with continuous perfusion; then, the expression of Runx2 and osteocalcin was increased [26]. Li et al. reported that the proliferation rates of human mesenchymal stem cells increased after loading fluid flow for 24 h, and gene expression of osteocalcin and osteopontin showed an increase [27]. In addition to providing support for the construction of tissue engineered bone tissue in vitro, these findings can also provide a theoretical basis for stem cells therapy to repair bone defects in vivo.

Various studies confirmed that Wnt2 was an extracellular activator of the W-β signaling pathway [28]. Some studies indicated that Wnt2 is closely related to osteogenesis. The mRNA levels of Wnt2 were higher in osteoblasts compared to their progenitors [29]. When compared to adjacent non-cancerous tissues, the expression of Wnt2 protein was elevated in human osteosarcoma tissues. It was also seen that there was a marked increase in expression in MG63 OS cell line in comparison with the human osteoblast hFOB 1.19 cell line [30]. Wnt2 in human dental follicle cells were significantly upregulated after induction by human dental follicle cells conditioned medium compared to human dental follicle cells in normal medium [31].

Some studies confirmed that Wnt2 were direct regulated by some microRNAs for controlling biological activities. miR-199a-5p has the ability to regulate myogenesis by suppressing Wnt signaling factors that play a role in balancing myogenic cell proliferation and differentiation by targeting FZD4, JAG1, and Wnt2 [32]. In the smooth muscle, MiR-199a-5p, which targets Wnt2, has a vital role in Wnt2-mediated regulation of proliferative and differentiation processes. It might act as a critical modulator of smooth muscle hypertrophy, which is vital for organ remodeling [33]. Promotion of proliferation of esophageal squamous cell carcinoma cell is brought about when miR-30a-3p/5p is regulated. This is brought about when Wnt2 and Fzd2 get inhibited, thereby activating the Wnt signaling pathway [34]. Furthermore, ectopic expression of miR-30a-3p significantly suppressed the migration, proliferation, and invasion of a human RCC cell line in vitro, while miR-30a-3p could inhibit tumor growth in vivo as well. Overexpression of miR-199a/b-5p, then inhibiting Wnt2, reduced autophagy, and induced cell apoptosis result in enhanced imatinib’s efficacy in K562R cells [35].

Various studies confirmed that Wnt7b was an extracellular activator and activator of the W-β signaling pathway [36]. Overexpression of Wnt7b in 1-month-old mice for 1 week markedly stimulated bone formation [37]. Out of the 19 Wnt ligands, it was found that Wnt7b was the most load-responsive during the formation of bone in aging C57Bl/6JN mice [38]. Wnt7b not only promotes bone formation through the W-β pathway but also in part through mTORC1 activation [39].

Some studies confirmed that some kinds of microRNA target Wnt7b for controlling biological activities. In human aortic smooth muscle cells, miR-29b mimic could target Wnt7b and potently repress Wnt7b/β-catenin protein expression, whereas miR-29b anti-miR had the ability to increase their expression. This indicated that miR-29b brings about negative regulation of Wnt7b/β-catenin signaling [40]. Overexpression of miR-G-1 inhibited the expression of Wnt7b, and then inhibiting cell proliferation, cell cycle progression, migration, invasion, and drug resistance in cervical cancer cells [41].

Some studies have proofed the miR-503-3p expression changed in many types of ocological lesions. It regulates p21 and CDK4 expression thereby inducing apoptosis of lung cancer cells [42]. miRNA expression profiles of lymphatic endothelial cells and functional analysis indicate that miR-503-3p might be as downstream targets of ELK3 in lymphatic endothelial cells, which cause to promote the migrating and invasive ability of breast cancer cells such as MDA-MB-231, Hs578T, and BT20 in vitro [43]. miR-503-3p promotes epithelial-mesenchymal transition in breast cancer by directly targeting SMAD2 and E-cadherin [44]. miR-503-3p inhibited tumor growth via the regulation of cancer stem cell proliferation and self-renewal, and it may function as a stemness-attenuating factor via cell-to-cell communications [45]. It was upregulated in plasma from primary resistance patients of epidermal growth factor receptor tyrosine kinase inhibitors in patients of non-small cell lung cancer [46]. miR-503-3p also play a critical role in non-oncological disease. miR-503-3p were differentially expressed between diabetic kidney disease cases and type 1 diabetes mellitus patients controls [47]. miR-503-3p was significantly downregulated in rats with acute respiratory distress syndrome and acute lung injury, who acquired the treatment of bone marrow-derived mesenchymal stem cells [48]. The function of miR-503-3p in bone metabolism is still unknown. Our study is the first to verify a novel role and target of miR-503-3p during OD. It has the potential to become a new regulated target for bone regeneration. We hypothesize that the inhibition of miR-503-3p could promote the osteogenic differentiation of hASCs during the construction of tissue-engineered bone in vitro, shorten the time, and accelerate the bone deposition.

## Conclusion

In this study, we designed various experiments to examine the function of miR-503-3p during the process of hASCs OD induced by cyclic strain. Cyclic strain regulated the process of hASCs OD in vitro by downregulating the expression of miR-503-3p and upregulating the expression of Wnt2 and Wnt7b. Furthermore, by modulating miR-503-3p activity, we conclude that miR-503-3p inhibit the W-β pathway by targeting Wnt2 and Wnt7b, which then inhibit the OD of hASCs induced by cyclic strain in vitro.

## Data Availability

The datasets generated and/or analyzed during the current study are not publicly available but are available from the corresponding author upon reasonable request.

## Abbreviations

miRNAs: MicroRNAs
OD: Osteogenic differentiation
hASCs: Human adipose-derived stem cells
W-β: Wnt/β-catenin
siRNA: Small interfering RNA
RT-PCR: Quantitative real-time PCR
UTR: Untranslated region
RUNX2: Runt-related transcription factor 2
α-MEM: Alpha-modified Eagle’s medium
FBS: Fetal bovine serum
EX-Wnt2: Wnt2 expression plasmid
siWnt2: Wnt2 siRNA
EX-Wnt7b: Wnt7b expression plasmid
siWnt7b: Wnt7b siRNA
EX-Ctrl: Negative control expression plasmid
siR-Ctrl: Negative control siRNA
miR-Ctrl mimic: Mimic negative control of the miR-503-3p mimic
miR-Ctrl inhibitor: Inhibitor negative control of the miR-503-3p inhibitor
wt-Wnt2: Wnt2 3′UTR-wild type
mu-Wnt2: Wnt2 3′UTR-mutant
wt-Wnt7b: Wnt7b 3′UTR-wild type
mu-Wnt7b: Wnt7b 3′UTR-mutant
siβ-catenin: Short interfering RNAs that targeted β-catenin
ALP: Alkaline phosphatase
SPARC: Secreted protein acidic and cysteine-rich
GAPDH: Glyceraldehyde-3-phosphate dehydrogenase
TBS: Tris-buffer saline
F: Fold
MCM: miR-Ctrl mimic
R: RUNX2
A: ALP
S: SPARC
W7: Wnt7b
β: β-Catenin
BMSCs: Bone marrow mesenchymal stem cells
